# Gemcitabine Eliminates Double Minute Chromosomes from Human Ovarian Cancer Cells

**DOI:** 10.1371/journal.pone.0071988

**Published:** 2013-08-22

**Authors:** Lisa Yu, Yan Zhao, Chao Quan, Wei Ji, Jing Zhu, Yun Huang, Rongwei Guan, Donglin Sun, Yan Jin, Xiangning Meng, Chunyu Zhang, Yang Yu, Jing Bai, Wenjing Sun, Songbin Fu

**Affiliations:** 1 Laboratory of Medical Genetics, Harbin Medical University, Harbin, China; 2 Key Laboratory of Medical Genetics (Harbin Medical University), Heilongjiang Higher Education Institutions, Harbin, China; Enzo Life Sciences, Inc., United States of America

## Abstract

Double minute chromosomes are cytogenetic manifestations of gene amplification frequently seen in cancer cells. Genes amplified on double minute chromosomes include oncogenes and multi-drug resistant genes. These genes encode proteins which contribute to cancer formation, cancer progression, and development of resistance to drugs used in cancer treatment. Elimination of double minute chromosomes, and therefore genes amplified on them, is an effective way to decrease the malignancy of cancer cells. We investigated the effectiveness of a cancer drug, gemcitabine, on the loss of double minute chromosomes from the ovarian cancer cell line UACC-1598. Gemcitabine is able to decrease the number of double minute chromosomes in cells at a 7500X lower concentration than the commonly used cancer drug hydroxyurea. Amplified genes present on the double minute chromosomes are decreased at the DNA level upon gemcitabine treatment. Gemcitabine, even at a low nanomolar concentration, is able to cause DNA damage. The selective incorporation of double minutes chromatin and γ-H2AX signals into micronuclei provides a strong link between DNA damage and the loss of double minute chromosomes from gemcitabine treated cells. Cells treated with gemcitabine also showed decreased cell growth, colony formation, and invasion. Together, our results suggest that gemcitabine is effective in decreasing double minute chromosomes and this affects the biology of ovarian cancer cells.

## Introduction

Gene amplification is a form of genomic instability that is frequently seen in cancers, and it can manifest cytogenetically as homogeneously staining regions (HSRs) or double minute chromosomes (DMs) [1], [2], [3], [4]. DMs are autonomously replicating, acentric, and atelometric circular DNA ranging from hundreds of kilobases to a few megabases in size [5], [6], [7], [8], [9], [10]. In metaphase spreads stained with a DNA binding dye, DMs can be seen under the microscope as single or paired minute chromatin much smaller than the chromosomes.

As an extrachromosomal vehicle for the amplifications of genomic DNA sequences, DMs contribute to cancer formation and progression because oncogenes and multi-drug resistance genes are frequently present in the amplified sequences and the proteins they encode are often over-expressed [11]. Examples of genes amplified on DMs include *MYCN* in neuroblastoma [12], *C-MYC* in colon cancer cells [13], *EGFR* in gliomas [14], and *eIF-5A2* in ovarian cancer cells [15], and all of which when lost via DMs contributes to reversal of the cancer phenotype [12], [13], [14], [16]. Elimination of amplifications of oncogenes on DMs has also been shown to induce apoptotic cell death, cellular differentiation, and cellular senescence [13], [17], [18].

Many studies have contributed to our understanding of the mechanism of the loss of DMs from cancer cells. The loss of DMs has been demonstrated in many cancer cell lines [12], [13], [17], [19], [20], [21], [22]. Non-lethal low concentrations of hydroxyurea (HU) has first been found to increase the loss of DMs from mouse cells containing amplified DHFR [23], and was later found to have the same effect in mammalian cancer cells [13], [24]. The loss of DMs by low concentrations of HU can increase drug sensitivity [24] and reduce tumorigenicity of cancer cell lines [13]. Most importantly, the loss of DMs was contributed to their entrapment into micronuclei (MN) [13] and this entrapment can also be enhanced by low concentrations of HU [25], [26].

There are two models of MN formation: budding/nucleation in interphase and post-mitotic formation [27]. Limited evidence exists for the contribution of HU to MN formation by budding/nucleation [25]. A detailed study indicates HU can induce MN formation through the post-mitotic model [28]. In this model, HU induces the detachment of DMs from mitotic chromosomes such that aggregates of DMs are formed after mitosis at the next G1 phase of the cell cycle. After cells enter S phase, the DMs aggregates are surrounded by lamin protein to produce a replicable cytoplasmic MN [28]. The molecular mechanism of HU on MN formation has been investigated intensively in colon cancer cells containing DMs [26]. Low concentrations of HU causes DNA damage in the cell nucleus in S phase, detectable as γ-H2AX foci, but the signals do not significantly overlap with DMs chromatin. As the damage is repaired and cells progress through the cell cycle, most γ-H2AX signals are lost by metaphase while any signal that remain overlap with DMs chromatin. DMs with γ-H2AX signal were found to detach from anaphase chromosomes and form MN in the next G1 phase [26].

HU is an inhibitor that specifically inhibits the Ribonucleotide reductase (RNR). RNR is an important enzyme required for the *de novo* synthesis of deoxyribonucleoside triphosphates (dNTPs) in cells by converting ribonucleotides to deoxyribonucleotides [29], [30], [31]. Ribonucleotide reductase is encoded by two genes *RRM1* and *RRM2*, corresponding to the large R1 and small R2 subunit of the enzyme respectively. HU is an important inhibitor of this enzyme, specifically inhibiting the R2 subunit. RNR is not only important for maintaining dNTP supplies needed for DNA replication but also plays an important role in maintaining genome integrity [32].

Gemcitabine (2′, 2′-difluorodeoxycytidine, GEM) is a newer anticancer drug and it inhibits the R1 subunit of RNR. It has two functional properties. One is the direct interaction with the R1 subunit of RNR which decreases RNR function and thereby decreases the dNTPs level in cells. Many studies have indicated that *RRM1* expression level determines GEM sensitivity or resistance [33], [34], [35], [36], [37]. Since GEM is a deoxycytidine analog, the second property of GEM is that it can be modified by cellular enzymes to produce dFdCTP (2′, 2′-difluorodeoxycytidine-5′-triphsophate) which can be incorporated into newly replicated DNA resulting in chain termination [38]. GEM is used to treat various cancers such as non-small cell lung cancer (NSCLC), pancreatic cancer, bladder cancer, breast cancer, and ovarian cancer [39], [40], [41], [42], [43].

Ovarian cancer is one of the leading gynecological malignancies. Despite recent advances in the treatment of this cancer, more than half of advanced disease patients develop resistance to therapy, experience recurrence of disease, and eventually die because of these properties [44]. The standard treatment of ovarian cancer is surgery followed by carboplatin plus paclitaxel therapy, however many patients develop recurrent disease with resistance to platinum therapy [45]. Upon the approval of the use of GEM in the treatment of ovarian cancers in Europe, USA, and other countries in recent years, GEM is becoming a promising new drug in the treatment of ovarian cancers. Recently GEM has been shown to be effective in the treatment of ovarian cancers especially in the platinum resistant subclass, either used alone or in combination with other drugs [1], [40], [44], [45], [46].

DMs were found to be present in primary ovarian carcinoma samples and ascites from ovarian cancer patients, and in established ovarian cancer cell lines [15], [47], [48], [49], [50]. One study has found that in patients the frequency of ovarian carcinoma with DMs is as high as 88% [51]. One study has found that for ovarian cancer patients treated with low concentrations of HU, the number of DMs decreased in cancer cells from these patients [52]. Although HU can be taken orally or injected intravenously, it is a weak inhibitor of RNR with a half life of only 3.4 hours before being eliminated by the kidney [53]. In this study, we determined whether GEM can selectively decrease certain gene amplifications via DMs, the mechanisms of DMs decrease, and what value it has in the treatment of ovarian cancer. GEM could decrease the number of DMs in ovarian cancer cells as well as genes amplified on the specific DMs at a concentration 7500X lower than HU. With the addition of GEM, the number of MN increased, and genes carried on DMs are selectively incorporated into these MN. Even low concentrations of GEM could cause DNA damage in cells, and these damaged DNA are also selectively incorporated into MN. We propose that GEM can cause DNA damage on DMs, which when not repaired, are selectively incorporated into MN and lost from cells. This in turn affects the biology of the cancer cells, such that cells show decreased growth, decreased colony formation, and decreased invasion.

## Materials and Methods

### 1. Cell Lines and Culture Conditions

The ovarian cancer cell line UACC-1598 was a kind gift from Dr. Xin-Yuan Guan (University of Hong Kong) [15]. The UACC-1598-4 cell line was a clone of UACC-1598 selected for the stable maintenance of a high number of DMs. It was made by the serial dilution method. All cell lines were maintained in Roswell park memorial institute 1640 (RPMI1640) media (GIBCO, Carlsbad, CA, USA) supplemented with 10% fetal bovine serum (FBS). Cells were grown at 37°C in a humidified atmosphere of 5% CO_2_ and passaged every 2 to 3 days when they grew confluent.

### 2. Drug Treatment and Growth Analysis

HU and GEM were purchased from Sigma-Aldrich (Saint louis, MI, USA) and Lilly France (France) respectively and prepared according to the manufacturer’s recommendations. The HU stock solution was made in dimethyl sulfoxide (DMSO) and the GEM stock solution was made in 0.9% NaCl. The concentrations of HU used in experiments were 100 and 150 µM following published concentrations [23], [24], [26]. The concentrations of GEM used were 10 and 20 nM, which were different dilutions of its half maximal inhibitory concentration (IC_50_) in the cell lines used in this study. The concentration of gemcitabine used in this study was 3–6 fold lower than the standard *in vivo* dose given to patients (Lilly France). The concentrations 10 and 20 nM of GEM corresponds to 0.01 IC_50_ and 0.02 IC_50_ of GEM in the UACC-1598-4 cell line. The same concentrations correspond to 0.032, and 0.064 IC_50_ of GEM in the UACC-1598 cell line. Cells were passaged every 2 to 3 days and fresh compounds were added. The IC_50_ of GEM was calculated using CellTiter 96 AQ_ueous_ One Solution Cell Proliferation Assay Kit from Promega (Madison, WI, USA) following the manufacturer’s protocol.

### 3. MTS, Colony Formation, and Invasion Assays

Briefly, the cell biological assays were carried out as follows. For MTS assay, 2000 cells were seeded into each well of 96-well plates. Cells were either allowed to grow in media alone or media containing 150 µM HU or 20 nM GEM. The OD of each well was read every day following the CellTiter 96 AQ_ueous_ One Solution Cell Proliferation Assay kit protocols and plotted. For colony formation, 2000 cells were seeded into each well of a 6-well plate either in the presence or absence of compounds. Cells were allowed to grow for 14 days before being washed with phosphate buffered saline (PBS), ethanol fixation, and Giemsa staining. Pictures were taken for each well and colonies were manually counted with ImageJ software. Three replicates were done for each condition. For cell invasion, sixty thousand cells were seeded in BD BioCoat Matrigel Invasion Chambers (BD Biosciences, Franklin Lakes, NJ, USA), and allowed to invade through a matrigel membrane. After 72 hours, the number of cells which invaded through the membrane was counted. Three replicates were performed for each compound condition.

### 4. Metaphase Spread

Cells were treated with Colcemid (Sigma) for 1.5 h, trypsinized, and washed in PBS before preparing metaphase spreads. Cells were first swelled in 9 ml of pre-warmed 0.075 M KCl for 14 min, at 37°C. After swelling, cells were pre-fixed with 1 ml of freshly made fixative solution (mixture of 3 methanol: 1 acetic acid) and immediately centrifuged. Cells were subsequently fixed and centrifuged twice with 10 mls of the fixative solution. Cell pellets from the last centrifugation were resuspended in 0.5 ml of fixative, and a small amount was taken in a Pasteur pipet and dropped onto clean glass slides. Mitotic chromosomes were observed by staining the slides with Giemsa and photographed with Olympus BX41 microscope (Olympus America Inc., Melville, NY, USA) connected to a JVC TK-C75U color video camera (JVC, Japan) at 1000X magnification. The number of DMs present in each cell was counted manually. The number of cells counted ranges from 60 to 100 for each sample.

### 5. Isolation of Genomic DNA and Real-time PCR

Cells were pelleted and genomic DNA was prepared with the QIAamp DNA Blood Mini Kit (QIAGEN, Germany) as described by the manufacturer. Real-time PCR was performed using the SYBR Premix Ex Taq II mix (TaKaRa Biotechnology (Dalian) Co., Ltd., China) in the LightCycler 480 (Roche Applied Science, Germany) Real-Time PCR detection system. The amplification level of *MYCN*, *EIF5A2*, and *MCL1* were detected, and *ACTB* served as the internal control. The mean ± standard deviation (SD) of three replicates was plotted for each set of comparisons between treatment and control cells. The primers used were as follows: *EIF5A2*, 5′-TACTTGGCAGAGATTAAACAGG-3′ (F), 5′-CAAAGTATTTGCACCTTGAAG-3′ (R); *MYCN*, 5′-ACCACAAGGCCCTCAGTAC-3′ (F), 5′-GCAACGGCATTCTCTCAG-3′ (R); *MCL1*, 5′-CTGGAGATTATCTCTCGGTAC-3′ (F), 5′-CTGACTCGTTTCGGTTTC-3′ (R); *ACTB*, 5′-CTTCTACAATGAGCTGCGTG-3′ (F), 5′-AAGCAAATAGAACCTGCAGAG-3′ (R).

### 6. Fluorescent *in situ* Hybridization (FISH)

Metaphase spreads were first prepared as described above and FISH was then performed with the following protocol. Bacterial artifical chromosome (BAC) clone RP11-654K19 for *EIF5A2*, BAC clone RP11-355H10 for *MYCN*, and BAC clone RP11-54A4 for *MCL1* were selected for FISH probes labeled with cyanine 3 (Cy3), fluorescein isothiocyanate (FITC), or cyanine 5 (Cy5). The slides were then counterstained with 4, 6-diamidino-2-phenylindole (DAPI) and photographs were taken with a Leica DM5000B microscope (Leica Microsystems, Germany) at 1000X magnification. The number of cells analyzed ranges from 180 to 280 for each sample.

The full signal of the cell nucleus we observed is set at 100%. The cells were grouped into 3 categories for the analysis of signal distribution: Group 1 includes cells with <30% signal, Group 2 includes cells with 30–60% signal, and Group 3 includes cells with >60% signal. The degree of the signal is quantified using MetaMorph software (Molecular Devices, Sunnyvale, CA, USA) for analyzing the relative area of the signal to the area of the cell nucleus. A micronucleus (MN) is defined as any DAPI stained region less bright than the DAPI stained nucleus and with an area of less than one third of the cell nucleus. Cells with two or more micronuclei were not counted to eliminate artifacts. Cells were also grouped based on MN status: Group A includes cells without MN, Group B includes cells with MN but the MN does not contain signal, and Group C includes cells with signal-containing MN.

### 7. Immunofluorescence

Cells were seeded on coverslips in six-well plates and treated with the different compounds for the indicated times before performing immunofluorescence. Briefly, cells on coverslips were washed 3×10 minutes in PBS, fixed with 4% paraformaldehyde for 20 minutes at room temperature, and rehydrated 3×10 minutes with PBS. The cells were permeabilized and blocked in KCM buffer (120 mM KCl, 20 mM NaCl, 10 mM Tris-HCl, 1 mM EDTA, 0.1% Triton X-100, 2% bovine serum albumin, and 10% skimmed milk powder) for 6 hours at 4°C and incubated with anti-phospho-H2AX (Ser139) antibody (clone JBW301, Millipore, Billerica, MA, USA) diluted in KCM buffer (1∶500) overnight at 4°C. Cells were then washed 3×10 minutes in PBS, incubated with CF555 goat anti-mouse IgG (H+L) (Biotium, Hayward, CA, USA) diluted in KCM buffer (1∶1000) for 1.5 hours at room temperature, and washed again for three times 10 minutes each with PBS. DNA was visualized by counterstaining the cells with DAPI and mounted on slides. Images were obtained using a Leica DM5000B microscope. To analyze the extent of DNA damage within cells, cells were grouped according to their γ-H2AX signals regardless of MN status. Cells were grouped into five categories including No signal, <30% signal, 30–60% signal, >60% signal, and full signal by MetaMorph software. For scoring of MN in cells released from compounds, cells were grouped into four categories: Group 1 contains cells without MN, Group 2 contains cells with MN and the MN does not contain signal, Group 3 includes cells with signal-containing MN, Group 4 includes cells with 1 or 2 strong signal spots within the cell nucleus near the periphery of the nucleus.

### 8. Statistical Analysis

The statistical significance in DMs data and real-time PCR data were analyzed using unpaired t-test with the exception of DMs data in UACC-1598-4, which were calculated with analysis of variance (*ANOVA*) followed by Dunnett’s multiple comparison post test. The statistical significance of FISH, γ-H2AX signal vs. no signal data and MN data were analyzed with *Fisher’s exact test*. Significance of MTS and colony formation data was calculated with *ANOVA*. The statistical significance of cell invasion was calculated with the *Chi-squared test*. Statistical significance in all data is denoted as follows: *denotes a *P* value of 0.01 to 0.05, **denotes a *P* value of 0.001 to 0.01, and ***denotes a *P* value of <0.001.

## Results

### 1. GEM Decreases the Number of DMs in the Ovarian Cancer Cell Line UACC-1598 and the Clone UACC-1598-4

**Figure 1:**
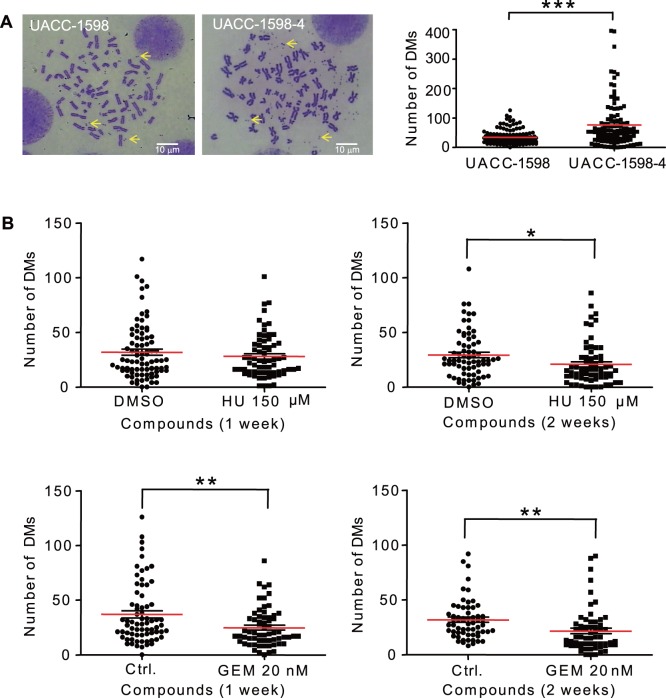
HU and GEM treatment decreases the number of DMs in UACC-1598. A. Representative pictures of metaphase spread of UACC-1598 and UACC-1598-4 cells. Arrows indicate DMs. The number of DMs in each metaphase cell was counted and plotted. ***indicates *P*<0.001. B. Metaphase spreads were prepared for cells grown in the presence of low concentrations of either HU or GEM for 1 week or 2 weeks. The number of DMs in each metaphase cell was counted. The red lines represent the mean and the black lines represent the SEM. *denotes a *P* value of 0.01 to 0.05 and **denotes a *P* value of 0.001 to 0.01.

UACC-1598 is an ovarian cancer cell line consisting of gene amplification in the form of DMs. UACC-1598-4 was created by the serial dilution method by seeding and isolating single cells from the parental cell line UACC-1598 and preparing metaphase spreads to determine the number of DMs a clone contains. This clone was found to have an increase in the number of DMs when compared with the parental cell line (Figure 1A). While the UACC-1598 cell line contained an average of 34.61 DMs per cell, UACC-1598-4 had an average of 76.16 DMs per cell which is more than double the value for the parental cell line. The difference between the number of DMs in UACC-1598 and the clone UACC-1598-4 were found to be statistically significant with a *P* value of <0.001.

We first tested whether the difference in the number of DMs between UACC-1598 and UACC-1598-4 affects sensitivity to GEM. We utilized the MTS method to calculate the IC_50_ of GEM in the two cell lines and found that there was a difference. The IC_50_ of GEM was 310 nM in UACC-1598 and 970 nM in UACC1598-4 (data now shown), and the difference is likely due to more DMs to be targeted and eliminated from UACC1598-4. Since the UACC1598-4 clone was selected for its generation and maintenance of a large number of DMs, we first used this cell line to determine if GEM can cause a loss of DMs because we hypothesized that it might be easier to detect a difference in the number of DMs. Cells were allowed to grow in media containing 10 nM (0.01×IC_50_) of GEM for two weeks before metaphase spreads were prepared. Our experiment indicated that the addition of low concentrations of GEM can decrease the number of DMs in UACC-1598-4 ovarian cancer cells, similar to the addition of HU (Figure S1A). The average number of DMs dropped from 76.16 to 25.23 in 150 µM of HU. In addition, the average number of DMs dropped to 48.99 in 10 nM of GEM.

We then determined whether GEM can decrease the number of DMs in the parental UACC-1598 cell line. Cells were allowed to grow in media containing 10 nM (0.032×IC_50_) and 20 nM (0.064×IC_50_) of GEM before metaphase spreads were prepared. We found that GEM at 10 nM was not effective in this cell line. Upon incubating UACC-1598 cells in 20 nM of GEM, we observed a statistically significant decrease in the number of DMs in both 1 week and 2 weeks after cells were continuously grown in media containing GEM (Figure 1B). The average number of DMs decreased from 37.00 to 24.83 after 1 week growth in the presence of GEM, and it decreased from 31.84 to 21.59 after 2 weeks of growth in GEM. Since HU was dissolved in DMSO, we eliminated the effect of DMSO in our experiments by comparing HU treated cells to DMSO treated cells. The average number of DMs decreased from 29.37 in cells grown in media containing DMSO to 20.77 in cells grown in media containing 150 µM HU for 2 weeks. We also compared control cells to DMSO treated cells and found that the difference between the two was not statistically significant (data not shown), indicating that DMSO at the concentration we used as the vehicle for HU does not significantly affect our experimental results. In addition, we also found that GEM is effective in decreasing DMs from other cell lines as well (data not shown). We therefore used HU at a concentration of 150 µM and GEM at a concentration of 20 nM for all our following experiments.

### 2. GEM Mediates the Loss of DMs Amplified Genes *EIF5A2* and *MCL1*

**Figure 2:**
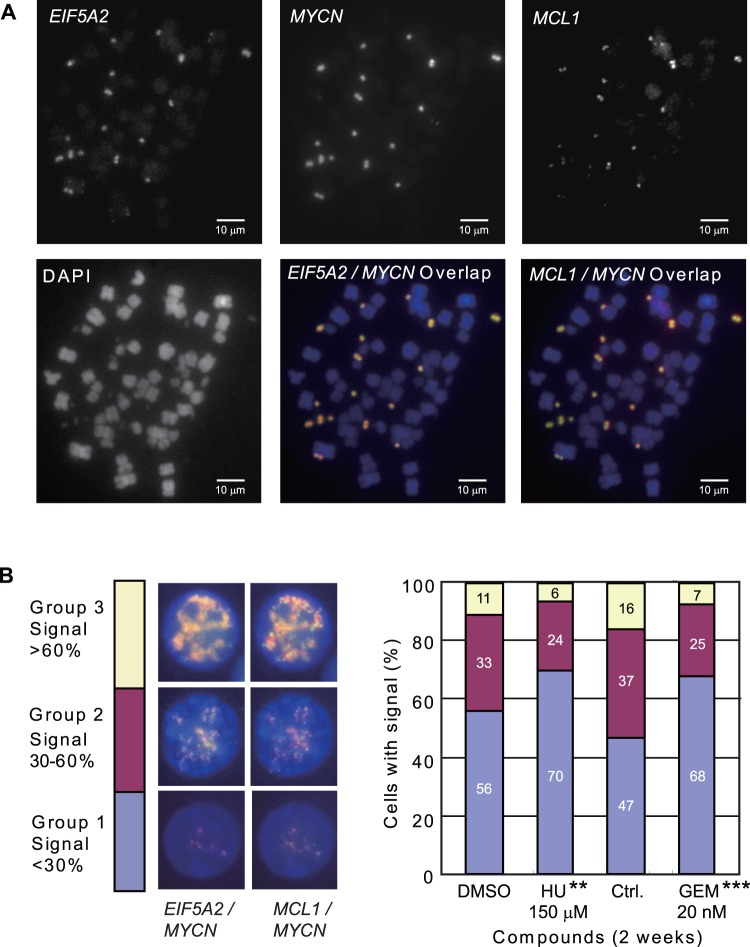
The signal of *EIF5A2*, *MYCN,* and *MCL1* genes is decreased in HU and GEM treated UACC-1598. A. Genes *EIF5A2*, *MYCN*, and *MCL1* are present on DMs in UACC-1598 cells. Metaphase spread of UACC-1598 cells detected with DNA probe for *EIF5A2*, *MYCN*, and *MCL1.* For the overlapped pictures, *EIF5A2* and *MCL1* signals are shown in red, and *MYCN* signal is shown in green. Overlapped *EIF5A2*/*MYCN* and *MCL1*/*MYCN* are shown in yellow. B. Representative pictures of *EIF5A2*/*MYCN* and *MCL1*/*MYCN* FISH signals in interphase cells of UACC-1598 and how individual cells are separated into different groups. *MYCN* signal is shown in green, *EIF5A2* and *MCL1* shown in red, and the overlap is shown in yellow. The percentage of cells in Groups 1, 2, and 3 is based on *EIF5A2*/*MYCN* and *MCL1*/*MYCN* FISH signals. The statistical analysis showed the differences of cells in Group 1 and Group 2/3 compared to the control cells. *denotes a *P* value of 0.01 to 0.05 and **denotes a *P* value of 0.001 to 0.01.

Once we found that GEM can decrease the number of DMs in both UACC-1598 and UACC-1598-4, the next question we asked is whether genes amplified on the DMs are also decreased. It is known that oncogenes *EIF5A2* and *MYCN* are two genes amplified in UACC-1598 [15], [16], and *MCL1* was also found to be amplified in this cell line (unpublished data). We first verified that these three genes we analyzed are present on DMs with FISH experiments (Figure 2A). We then separated cells grown in HU or GEM into 3 groups according to the level of *EIF5A2, MYCN,* and *MCL1* signals (Figure 2B). Group 1 includes cells with <30% signal, which corresponds to cells with less signals from *EIF5A2*, *MYCN*, and *MCL1*, and is an indicator of cells with less number of DMs; group 2 includes cells with 30–60% signal; and group 3 includes cells with >60% signal, which is an indicator of cells with more DMs. The percentage of cells in each group was plotted. The percentage of cells in Group 3, which contains cells with greater than 60% signals, decreased in the presence of either HU (from 11% to 6%) or GEM (from 16% to 7%). In addition, the percentage of cells in Group 2 decreased from 33% to 24% for HU treated cells and 37% to 25% in GEM treated cells. In contrast to the decrease in the percentages of cells in Group 2 and 3, the percentage of cells in Group 1 increased 14 to 21% in cells treated with HU (from 56% to 70%) or GEM (from 47% to 68%). The increase in the percentage of cells in group 1 in the HU or GEM treated cells indicates cells have started losing their DMs. In addition, we consolidated groups 2 and 3 into one large group, and statistical analysis showed that the differences observed between DMSO and HU or control and GEM treated cells is statistically significant (Figure 2B). These results indicate that the signal of the amplified genes *MYCN*, *EIF5A2*, and *MCL1* on DMs in UACC-1598 cells decreased in the presence of both HU and GEM. A similar result was seen for GEM when analyzing *MYCN* and *EIF5A2* in UACC-1598-4 cells (Figure S1B).

**Figure 3:**
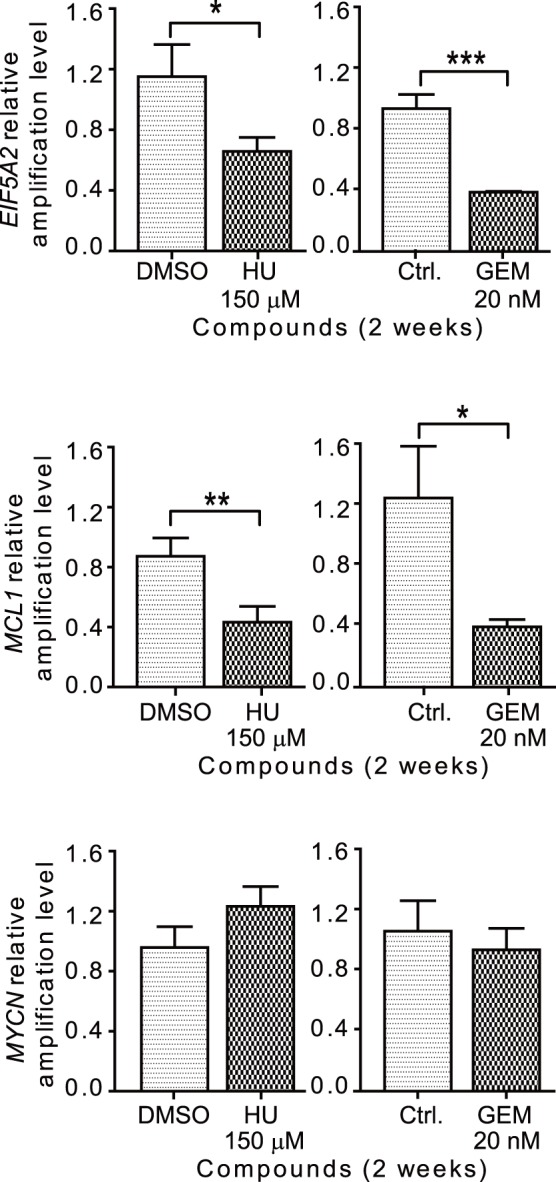
The amplification of genes present on DMs is decreased in UACC-1598 cells grown in the presence of HU and GEM by real-time PCR analysis. The amplification level of genes *EIF5A2*, *MCL1*, and *MYCN* was analyzed by real-time PCR. The amplification level of each gene in compound treated cells is compared to control cells (DMSO for HU treated, and Ctrl. for GEM treated), and the mean relative amplification level ± SD is graphed. *denotes a *P* value of 0.01 to 0.05 and **denotes a *P* value of 0.001 to 0.01, ***denotes *P*<0.001.

Furthermore, we also confirmed our results by performing real-time PCR to analyze the amplification of *EIF5A2, MYCN,* and *MCL1* in cells grown in the presence of HU or GEM (Figure 3). We saw a statistically significant decrease in the amplification of *EIF5A2* and *MCL1* genes when cells were grown in the presence of either HU or GEM. For cells treated with HU, a decrease in relative DNA amplification level of 1.151±0.213 to 0.658±0.092 was observed for *EIF5A2*, and a decrease in amplification level of 0.860±0.122 to 0.422±0.106 was observed for *MCL1*. For cells treated with GEM, a decrease in amplification level of 0.930±0.094 to 0.383±0.005 was observed for *EIF5A2*, and a decrease in amplification level of 1.242±0.343 to 0.388±0.048 was observed for *MCL1*. However, we did not detect a statistically significant decrease in the amplification of *MYCN* in either HU or GEM treated cells. Similar results were also acquired for UACC-1598-4 cells, although with obscure change of *MCL1* (Figure S1C).

### 3. Entrapment of DMs Amplified Genes in Micronuclei

**Figure 4:**
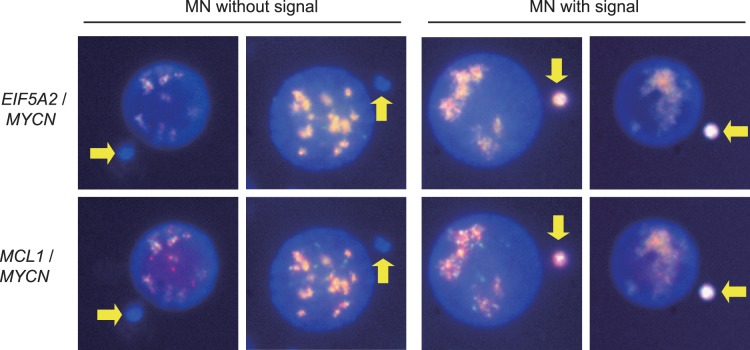
Entrapment of *EIF5A2, MYCN* and *MCL1* in MN in UACC-1598 ovarian cancer cell line by growth in HU and GEM. The left two panels are representative pictures of cells showing MN without signal and the right two panels are representative pictures of cells showing MN with *EIF5A2*/*MYCN* or *MCL1*/*MYCN* signal. *MYCN* signal is shown in green, *EIF5A2* and *MCL1* are shown in red, and the overlap is shown in yellow. Arrows indicate MN.

One way in which DMs are thought to be eliminated from cells is by incorporation into micronuclei. With amplified genes as probes, we observed some cells with MN containing probes while others have MN not containing the probes (Figure 4). We determined the percentage of MN formation in cells treated with HU or GEM, and also observed whether these micronuclei contained *EIF5A2, MYCN* and *MCL1* signals (Table 1). Vehicle DMSO treated cells have a MN formation frequency of 13.87×10^−2^ while HU treated cells have a MN formation frequency of 30.61×10^−2^, indicating HU is effective at inducing MN formation. In addition, GEM is also effective at inducing MN formation. Cells treated with 20 nM GEM have a MN formation frequency of 21.82×10^−2^ compared to 13.98×10^−2^ in control cells. Upon examining *EIF5A2, MYCN,* and *MCL* signals in the MN, we also noticed an increase in the frequency of MN which contains *EIF5A2, MYCN,* and *MCL* signals, designated MN (*EIF5A2*+ *MYCN*+ *MCL+*) or MN (+) for short, for cells in the HU and GEM treatment groups. A 2.57 and 1.81 fold increase in MN (+) frequency was observed for HU and GEM treated cells respectively. Together, our FISH results suggest that DMs amplified genes *EIF5A2, MYCN,* and *MCL* are lost from cells treated with either HU or GEM by being selectively entrapped into MN. Similar results were obtained with UACC-1598-4 cells (Table S1).

**Table 1:** Induction of MN and MN (*EIF5A2+ MYCN+ MCL1+*) by HU and GEM.

	Total cell number	Cells with MN	MN frequency (×10^−2^)	Fold change	Cells with MN (+)	MN (+) frequency (×10^−2^)	Fold change
DMSO	274	38	13.87	1.00	25	9.12	1.00
HU (150 µM)	196	60	30.61***	2.21	46	23.47***	2.57
Ctrl.	279	39	13.98	1.00	19	6.81	1.00
GEM (20 nM)	275	60	21.82*	1.56	34	12.36*	1.81

MN (+) indicates cells with *EIF5A2*, *MYCN*, and *MCL1* signals in the MN.

* denotes a *P* value of 0.01 to 0.05, and ***denotes a *P* value of <0.001.

### 4. Low Concentrations of GEM causes DNA Damage in Cells Similar to Low Concentrations of HU

**Figure 5:**
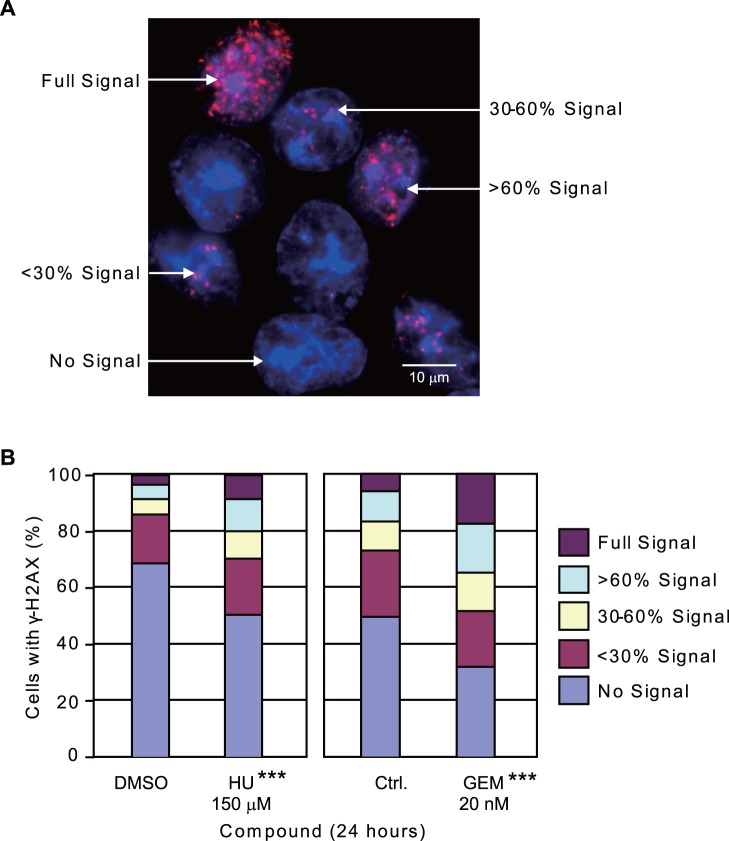
Induction of γ-H2AX foci by HU and GEM in the UACC-1598 ovarian cancer cell line. A. Representative picture of γ-H2AX foci detected by immunofluorescence and examples of how cells are grouped into different categories based on the degree of DNA damage. DAPI stained DNA is shown in blue and immunofluorescence of γ-H2AX foci is shown in red. B. Percentage of cells in each category based on amount of γ-H2AX foci. The statistical analysis showed the differences of cells in the No signal group vs. all other groups compared to the control cells. ***denotes a *P* value of <0.001.

Low concentrations of HU have been found to cause DNA damage detectable as γ-H2AX foci in cells. We determined whether GEM can also cause DNA damage. We treated UACC-1598 cells with HU or GEM for 24 hours and performed immunofluorescence to determine the amount of γ-H2AX foci in individual cells. We grouped cells into five categories based on the amount of signal each cell contains (Figure 5A) and found that cells treated with GEM show increased γ-H2AX foci similar to cells treated with HU (Figure 5B). The percentage of cells with γ-H2AX foci increased from 31.15 to 49.33 when treated with HU while the percentage of cells with γ-H2AX foci increased from 50.54 to 67.98 when treated with GEM. In addition, we also performed statistical analysis for cells with no γ-H2AX signal compared to cells with γ-H2AX signal and found that both 150 µM HU and 20 nM GEM treated cells showed statistical significance with respect to their respective controls (Figure 5B). We further confirmed this result in the clone UACC-1598-4 cell line (Figure S1D). This result indicates GEM at low concentrations is also effective at inducing γ-H2AX foci formation in cells.

### 5. GEM Induces the Generation of Micronuclei Containing Bright Uniform γ-H2AX Signals

**Figure 6:**
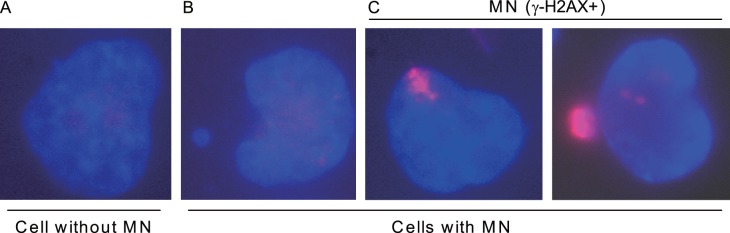
Classification of cells into different categories based on the presence of MN and γ-H2AX signal within MN. DAPI stained DNA is shown in blue, and γ-H2AX immunofluorescence signal is shown in red. The different groups: A, Cells without MN, B, Cells with MN, and C, Cells with MN (γ-H2AX+) are indicated.

Studies have also indicated that DNA damage induced by HU can facilitate the formation of MN containing γ-H2AX signals [26], [54]. It is hypothesized that these MN selectively incorporates damaged DNA, and the incorporated DNA are then lost from cells. We incubated UACC-1598 cells in different compounds for 24 hours, washed away the compounds, released the cells into media without compounds for 24 and 48 hours, and detected MN frequency together with γ-H2AX signals (Figure 6). We found that 24 hours after release from GEM treatment, there was a statistically significant increase in MN frequency (from 4.87×10^−2^ to 10.36×10^−2^, a 2.13 fold increase) when compared to control cells (Table 2). This is in agreement with cells released from HU, in which the MN frequency showed a statistically significant increase from 4.65×10^−2^ to 8.44×10^−2^ (a 1.82 fold increase). We detected γ-H2AX by immunofluorescence and when we analyzed the frequency of MN in which there was bright uniform γ-H2AX signal, designated MN (γ-H2AX+) or MN (+) for short, we observed a statistically significant increase (from 3.06×10^−2^ to 7.10×10^−2^, a 2.32 fold increase for HU; from 4.10×10^−2^ to 7.32×10^−2^, a 1.79 fold increase for GEM) for cells released from HU or GEM when compared to control cells. A similar trend was seen in a repeat experiment in UACC-1598-4 (Table S2 and S3). For 48 hours after release, a similar trend is seen in MN and MN (+) frequency albeit some with lower levels of fold change when compared with their respective controls (Table 3). Our results show that after cells are released from GEM treatment, a significant proportion of cells accumulate MN with bright γ-H2AX staining.

**Table 2:** Induction of MN and MN (γ*-*H2AX*+*) by HU and GEM (24 hours after release).

	Total cellnumber	Cells with MN	MN frequency (×10^−2^)	Fold change	Cells with MN (+)	MN (+) frequency (×10^−2^)	Fold change
DMSO	947	44	4.65	1.00	29	3.06	1.00
HU (150 µM)	972	82	8.44**	1.82	69	7.10**	2.32
Ctrl.	904	44	4.87	1.00	37	4.10	1.00
GEM (20 nM)	724	75	10.36**	2.13	53	7.32**	1.79

MN (+) indicates cells with γ-H2AX immunofluorescence signals in the MN.

** denotes a *P* value of 0.001 to 0.01.

**Table 3:** Induction of MN and MN (γ*-*H2AX*+*) by HU and GEM (48 hours after release).

	Total cellnumber	Cells with MN	MN frequency (×10^−2^)	Fold change	Cells with MN (+)	MN (+) frequency (×10^−2^)	Fold change
DMSO	821	53	6.46	1.00	40	4.87	1.00
HU (150 µM)	837	84	10.04*	1.55	63	7.53*	1.54
Ctrl.	982	54	5.50	1.00	33	3.36	1.00
GEM (20 nM)	670	81	12.09**	2.20	52	7.76**	2.31

MN (+) indicates cells with γ-H2AX immunofluorescence signals in the MN.

* denotes a *P* value of 0.01 to 0.05, and **denotes a *P* value of 0.001 to 0.01.

### 6. GEM Affects the Biological Properties of Ovarian Cancer Cells

**Figure 7:**
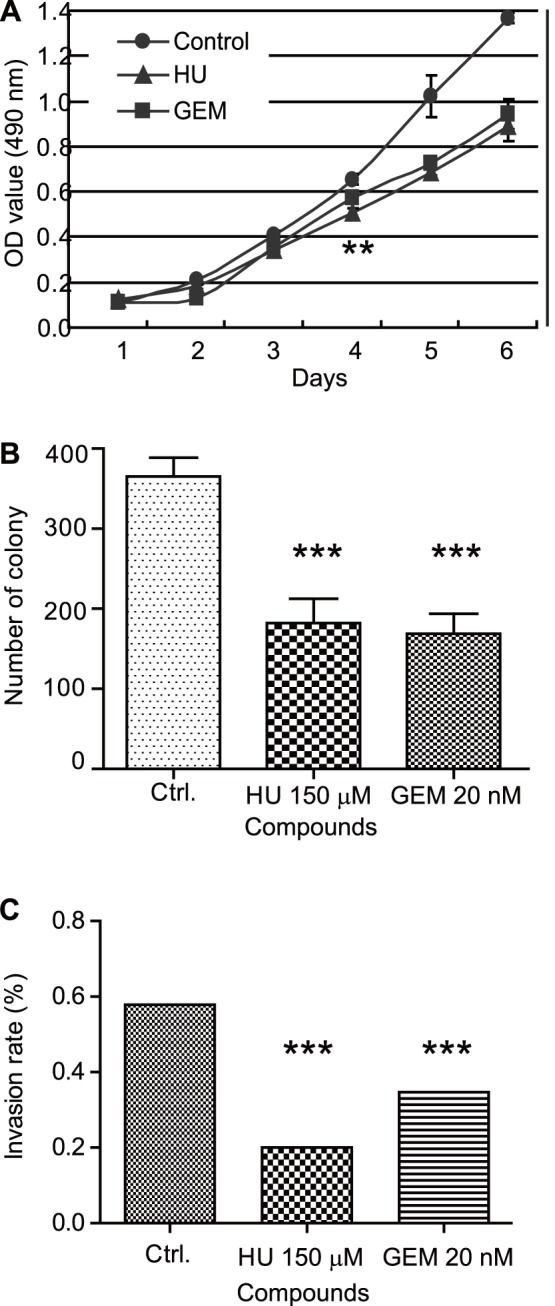
Treatment with HU or GEM decreases the malignancy of UACC-1598 ovarian cancer cells. A. The growth of UACC-1598 cells is decreased when grown in the presence of HU or GEM. The OD value of cells were measured every day for 6 days and plotted as mean ± SD. **denotes a *P* value of 0.001 to 0.01 from day 4 onwards when compared to the control group. B. Colony formation of UACC-1598 cells is decreased when grown in the presence of HU or GEM. The average numbers of colonies ± SD decreased from 365±24 colonies to 182±30 for HU treated cells and 168±25 for GEM treated cells. ***denotes a *P* value of <0.001 when compared to the control group. C. One representative trial of cell invasion is plotted for Ctrl., HU treated, and GEM treated cells. The invasion percentage of cells decreased from 0.58% for control group to 0.20% for HU treated cells and 0.35% for GEM treated cells. ***denotes a *P* value of <0.001 when compared to the control group.

We also tested whether addition of low levels of GEM affects the biological properties of ovarian cancer cells. By using the MTS assay, we observed a significant decrease from day four onward for UACC-1598 cells grown in the presence of either 150 µM HU or 20 nM GEM (Figure 7A). In addition, we also observed a significant decrease in colony formation for cells treated with either of the two compounds. The average number of colonies from the same plating number decreased from 365±24 colonies to 182±30 for HU treated cells and 168±25 for GEM treated cells (Figure 7B). Furthermore, we also observed a decrease in invasion for HU and GEM treated cells. The percentage of cells invading through the membrane for one experiment is plotted, which decreased from 0.58% for control group to 0.20% for HU treated cells and 0.35% for GEM treated cells (Figure 7C). Two other repeats show similar results. The percentage of cells invading through the membrane decreased from an average of 42% for control cells to 23% and 24% for HU and GEM treated cells respectively (data not shown). Taken together, these data shows that in addition to affecting double minute chromosomes, GEM also affects the biological properties of ovarian cancer cells.

## Discussion

GEM is a newer anticancer drug and it could inhibit the R1 subunit of RNR. It is used to treat various cancers such as NSCLC, pancreatic cancer, bladder cancer, breast cancer, and ovarian cancer [39], [40], [41], [42], [43]. Here our studies show that GEM is a compound that could reduce the number of DMs from ovarian cancer cell lines at low nanomolar concentrations. This is 7500X more effective than HU, which is effective at µM level, in decreasing DMs from ovarian cancer cell lines and other cancer and drug resistant cell lines as previous reported [23], [24], [26]. However, this does not mean inhibiting the R1 subunit of RNR is better than inhibiting R2. In addition to the fact that they together make up the functional RNR, R1 and R2 also have other separate functions from each other. We cannot know if inhibiting one will be better than the other. Functional complementation experiments will have to be performed in order to determine this for sure.

In addition, our studies report that the amplification levels of genes present on DMs decreased when UACC-1598 cells were treated with HU and GEM. We confirmed this by two independent methods, FISH analysis and real-time PCR technique. We observed different levels of decrease in the individual amplified genes, *EIF5A2*, *MYCN* and *MCL1*. Previous studies have shown that low concentrations of HU accelerated the loss of extrachromosomally amplified *DHFR* from mouse MTX resistance cells or other oncogenes from mammalian cancer cells [13], [23], [24]. These results suggest that there is selectivity in which individual DMs or individual amplified genes on DMs are lost from a cell. Since DMs exist as a mixed heterogeneous population in an individual cell, and there are also differences between cells in a population, we can conclude that GEM is able to cause a decrease in the amplification level of certain amplified genes which are located on DMs from cancer cells.

Previous studies have shown that DMs are selectively incorporated into MN in HU treated cells and this may facilitate the loss of DMs from cells [13], [25], [26]. We investigated whether this is also the case for GEM treated cells, and our results provide convincing evidence that treatment with GEM facilitates the incorporation of DMs into MN with FISH analysis. The mechanism by which DMs are lost from the cell once they are captured within MN remains to be elucidated. It is possible that MN may be lost from cells by being secreted into the growth media, targeted for fusion with phagosomes, or reincorporated into the cell nucleus [26], [55]. Together with our observation that there is a decrease in the number of DMs after cells are grown in media containing GEM, the most likely reason for the incorporation of DMs in MN is as an intermediate step to prepare for their loss from cells.

 Why DMs are selectively captured into MN? Our studies provide further explanation of this interesting phenomenon. That is, the DNA damage acquired in HU and GEM treated cells contributes to the loss of DMs. One similarity between HU and GEM is that they target either R2 or R1 subunit of the same protein complex, RNR, which is needed for the *de novo* synthesis of dNTPs. One reason for the selective loss of DMs has been linked to DNA damage accumulation for cells grown in the presence of HU [26], and we confirmed that this is also the case for cells grown in the presence of GEM. Previous studies have shown that DNA damage occurs in cells treated with a high concentration of GEM [56]. We provided evidence here that DNA damage also occurs, as detected by the formation of γ-H2AX foci, in the presence of low concentration of GEM similar to low concentrations of HU. Our study provides further evidence that any agent that causes cells to accumulate DNA damage is very likely to cause a decrease in DMs numbers. Here, we have also shown that there was a significant accumulation of cells with MN containing bright γ-H2AX signals when cells were released from GEM treatment into fresh media and allowed to repair the DNA damage (Figure 5, Table 2 and 3). At 24 and 48 hours after release from GEM, the accumulation of bright γ-H2AX signal in MN is more prevalent 48 hours after release from GEM. However, there was a decrease in γ-H2AX signal within the cell nucleus both 24 and 48 hours after release from GEM compared to cells before release from GEM treatment (data not shown), suggesting that DNA damage is being repaired in the cells and that damaged DNA that is not being repaired is selectively incorporated into MN. In addition, the frequency of accumulation of MN (γ-H2AX+) is comparable to the frequency of accumulation of MN (*EIF5A2*+ *MYCN*+ *MCL*+). Shimizu group reported that low-dose HU induced many γ-H2AX foci throughout the nucleus in S-phase, but the signal rarely overlapped with DMs [26]. As the damage was repaired and cell progressed through the cell cycle, most chromosomal γ-H2AX foci were lost by metaphase, whereas, those that persisted frequently associated with DMs. Those DMs were always aggregated at metaphase and remained behind the separating chromatids during anaphase, thus generating MN. Together with previous data, our study provides a strong link between DMs loss and DNA damage accumulation in MN.

Another important observation from our studies is that GEM affects the biological properties of ovarian cancer cells. The observed decrease in cell growth, colony formation and cell invasion is indicative of a decrease in malignancy of the ovarian cancer cells when treated with GEM. The presence of DMs has important implications for the malignant phenotype, the decrease in the number of DMs as well as the amplified genes might result in the reversal of cancer phenotype, by inducing proliferation arrest or increase apoptotic cell death [12], [13], [17], [20], [57]. Our results also provide a direct relationship between the loss of DMs or genes located on DMs and a decrease in ovarian cancer cell malignancy. Our study therefore sets the foundation for future research into the mechanism of DMs loss from cells, and for future research into the effectiveness of the use of GEM in the treatment of the subclass of ovarian cancer which contains DMs.

## Supporting Information

Figure S1 **HU and GEM treatment decreases the number of DMs and oncogenes amplified on DMs and causes DNA damage detectable as γ-H2AX foci.** A. The number of DMs in each metaphase cell in UACC-1598-4 was counted and plotted for control cells or treated cells. Solid red line denotes the mean. ***indicates *P*<0.001 when compared with the control group. B. Quantification and statistical analysis of cells in Group 1 and Group 2/3 in UACC-1598-4 cells according to guidelines in Figure 2. *denotes a *P* value of 0.01 to 0.05 when compared with the control group. C. The amplification of oncogenes present on DMs is decreased in UACC-1598-4 cells grown in the presence of HU and GEM by real-time PCR analysis. D. Quantification of cells in the No signal group vs. γ-H2AX groups for cells treated with HU or GEM. Statistical significances are as indicated where *denotes a *P* value of 0.01 to 0.05 and ***denotes a *P* value of <0.001 when compared with the control group.
(PDF)

Table S1 MN and MN (*EIF5A2+ MYCN+ MCL1*+) frequency of HU and GEM treated UACC-1598-4
(DOC)

Table S2 MN and MN (γ*-*H2AX*+*) frequency of HU and GEM treated UACC-1598-4 (24 hours after release)
(DOC)

Table S3 MN and MN (γ*-*H2AX*+*) frequency of HU and GEM treated UACC-1598-4 (48 hours after release)
(DOC)
