# Supplementary figures and images for: Gemcitabine Eliminates Double Minute Chromosomes from Human Ovarian Cancer Cells
Source: PLoS One. 2013 Aug 22;8(8):e71988. doi: 10.1371/journal.pone.0071988 (PMC3750019; doi:10.1371/journal.pone.0071988)

Figure S1

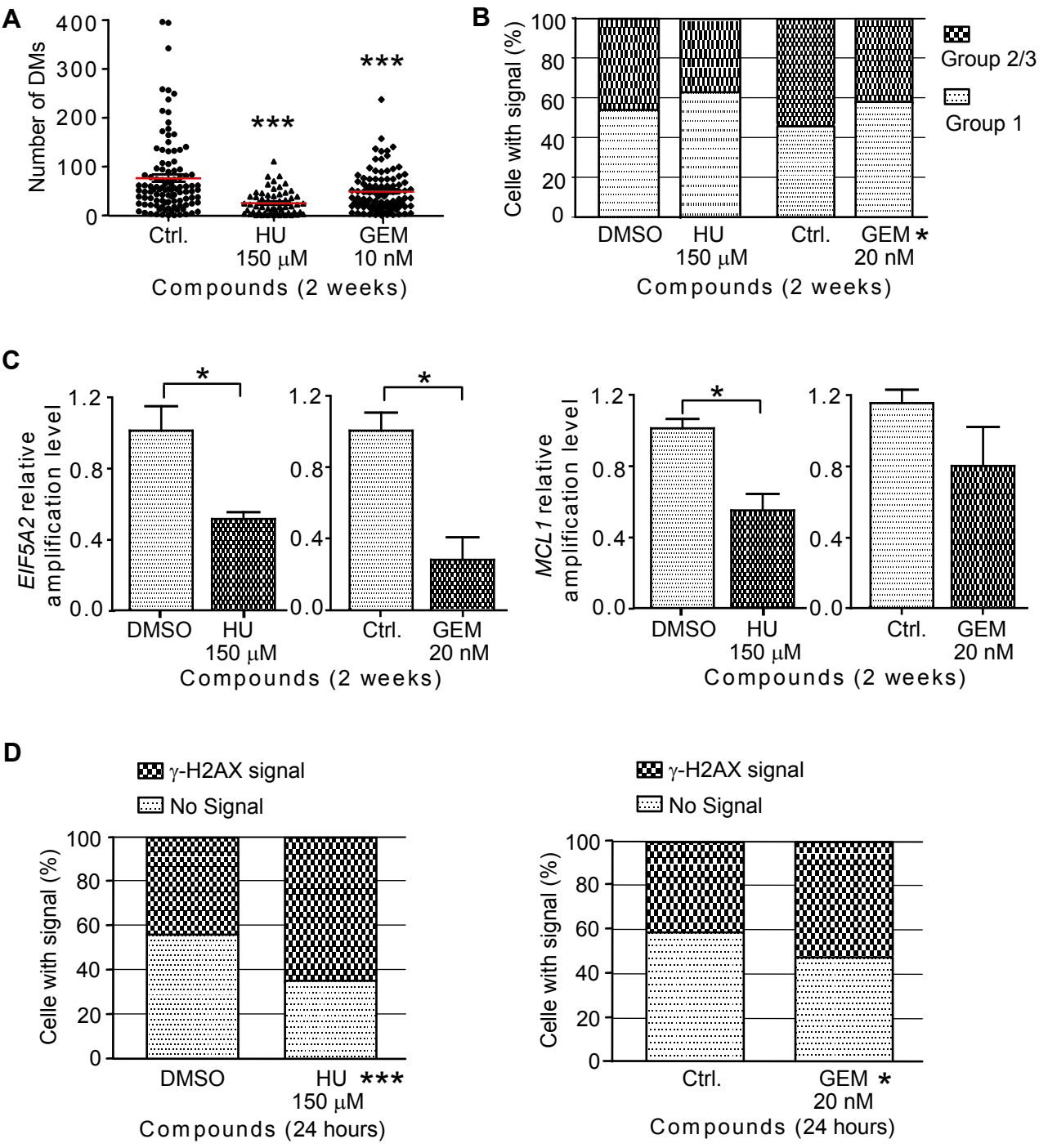

Supplement: Figure S1 — HU and GEM treatment decreases the number of DMs and oncogenes amplified on DMs and causes DNA damage detectable as γ-H2AX foci. A. The number of DMs in each metaphase cell in UACC-1598-4 was counted and plotted for control cells or treated cells. Solid red line denotes the mean. ***indicates P<0.001 when compared with the control group. B. Quantification and statistical analysis of cells in Group 1 and Group 2/3 in UACC-1598-4 cells according to guidelines in Figure 2. *denotes a P value of 0.01 to 0.05 when compared with the control group. C. The amplification of oncogenes present on DMs is decreased in UACC-1598-4 cells grown in the presence of HU and GEM by real-time PCR analysis. D. Quantification of cells in the No signal group vs. γ-H2AX groups for cells treated with HU or GEM. Statistical significances are as indicated where *denotes a P value of 0.01 to 0.05 and ***denotes a P value of <0.001 when compared with the control group. (PDF) [file pone.0071988.s001.pdf]
