# Supplementary material for: Gemcitabine Eliminates Double Minute Chromosomes from Human Ovarian Cancer Cells
Source: PLoS One. 2013 Aug 22;8(8):e71988. doi: 10.1371/journal.pone.0071988 (PMC3750019; doi:10.1371/journal.pone.0071988)
Supplement: Table S1 — MN and MN (EIF5A2+ MYCN+ MCL1+) frequency of HU and GEM treated UACC-1598-4 (DOC) [file pone.0071988.s002.doc]

**Table S1. MN and MN (*EIF5A2+* *MYCN+ MCL1***+) frequency of HU and GEM treated UACC-1598-4

|  | Total cell number | Cells with MN | MN frequency (x10^-2^) | Fold change | Cells with MN (+) | MN (+) frequency (x10^-2^) | Fold change |
| --- | --- | --- | --- | --- | --- | --- | --- |
| DMSO | 130 | 11 | 8.46 | 1.00 | 9 | 6.92 | 1.00 |
| HU (150 µM) | 106 | 24 | 22.64** | 2.67 | 19 | 17.92* | 2.59 |
| Ctrl. | 225 | 17 | 7.56 | 1.00 | 10 | 4.44 | 1.00 |
| GEM (20 nM) | 170 | 32 | 18.82** | 2.49 | 18 | 10.59* | 2.38 |

* denotes a *P* value of 0.01 to 0.05, and ** denotes a *P* value of 0.001 to 0.01.
