# Supplementary material for: Gemcitabine Eliminates Double Minute Chromosomes from Human Ovarian Cancer Cells
Source: PLoS One. 2013 Aug 22;8(8):e71988. doi: 10.1371/journal.pone.0071988 (PMC3750019; doi:10.1371/journal.pone.0071988)
Supplement: Table S2 — MN and MN (γ-H2AX+) frequency of HU and GEM treated UACC-1598-4 (24 hours after release) (DOC) [file pone.0071988.s003.doc]

**Table S2 MN and MN (γ*-*H2AX*+*) frequency of HU and GEM treated UACC-1598-4 (**24 hours after release)

|  | Total cell number | Cells with MN | MN frequency (x10^-2^) | Fold change | Cells with MN (+) | MN (+) frequency (x10^-2^) | Fold change |
| --- | --- | --- | --- | --- | --- | --- | --- |
| DMSO | 601 | 75 | 12.48 | 1.00 | 52 | 8.65 | 1.00 |
| HU (150 µM) | 502 | 89 | 17.73* | 1.42 | 38 | 7.57 | 0.88 |
| Ctrl. | 751 | 48 | 6.39 | 1.00 | 34 | 4.53 | 1.00 |
| GEM (20 nM) | 462 | 88 | 19.05*** | 2.98 | 63 | 13.64*** | 3.01 |

MN (+) indicates cells with γ-H2AX immunofluorescence signals in the MN. * denotes a *P* value of 0.01 to 0.05, and *** denotes a *P* value of <0.001.
