# Supplementary material for: Gemcitabine Eliminates Double Minute Chromosomes from Human Ovarian Cancer Cells
Source: PLoS One. 2013 Aug 22;8(8):e71988. doi: 10.1371/journal.pone.0071988 (PMC3750019; doi:10.1371/journal.pone.0071988)
Supplement: Table S3 — MN and MN (γ-H2AX+) frequency of HU and GEM treated UACC-1598-4 (48 hours after release) (DOC) [file pone.0071988.s004.doc]

**Table S3 MN and MN (γ*-*H2AX*+*) frequency of HU and GEM treated UACC-1598-4 (48 ho**urs after release)

|  | Total cell number | Cells with MN | MN frequency (x10^-2^) | Fold change | Cells with MN (+) | MN (+) frequency (x10^-2^) | Fold change |
| --- | --- | --- | --- | --- | --- | --- | --- |
| DMSO | 764 | 84 | 11.00 | 1.00 | 59 | 7.72 | 1.00 |
| HU (150 µM) | 672 | 109 | 16.22** | 1.47 | 46 | 6.85 | 0.89 |
| Ctrl. | 621 | 59 | 9.50 | 1.00 | 41 | 6.60 | 1.00 |
| GEM (20 nM) | 552 | 79 | 14.31* | 1.51 | 54 | 9.78 | 1.48 |

MN (+) indicates cells with γ-H2AX immunofluorescence signals in the MN. * denotes a *P* value of 0.01 to 0.05, and ** denotes a *P* value of 0.001 to 0.01.
